# Bone-Specific Metastasis Pattern of Advanced-Stage Lung Adenocarcinoma According to the Localization of the Primary Tumor

**DOI:** 10.3389/pore.2021.1609926

**Published:** 2021-09-23

**Authors:** Peter Radeczky, Judit Moldvay, Janos Fillinger, Beata Szeitz, Bence Ferencz, Kristiina Boettiger, Melinda Rezeli, Krisztina Bogos, Ferenc Renyi-Vamos, Konrad Hoetzenecker, Balazs Hegedus, Zsolt Megyesfalvi, Balazs Dome

**Affiliations:** ^1^ Department of Thoracic Surgery, National Institute of Oncology, Semmelweis University, Budapest, Hungary; ^2^ National Koranyi Institute of Pulmonology, Budapest, Hungary; ^3^ MTA-SE NAP, Brain Metastasis Research Group, Hungarian Academy of Sciences, Budapest, Hungary; ^4^ Department of Pulmonology, Semmelweis University, Budapest, Hungary; ^5^ Division of Oncology, Department of Internal Medicine and Oncology, Semmelweis University, Budapest, Hungary; ^6^ Department of Thoracic Surgery, Comprehensive Cancer Center, Medical University of Vienna, Austria; ^7^ Department of Biomedical Engineering, Lund University, Lund, Sweden; ^8^ Department of Thoracic Surgery, Ruhrlandklinik, University Clinic Essen, Essen, Germany; ^9^ 2nd Department of Pathology, Semmelweis University, Budapest, Hungary

**Keywords:** lung cancer, adenocarcinoma, metastasis pattern, bone metastasis, tumor localization

## Abstract

**Background:** Patients with advanced-stage lung adenocarcinoma (LADC) often develop distant metastases in the skeletal system. Yet, the bone-specific metastasis pattern is still controversial. We, therefore, aimed to examine how the primary tumor location affects bone specificity and survival in LADC patients diagnosed with skeletal metastases.

**Methods:** In total, 209 bone-metastatic Caucasian LADC patients from two thoracic centers were included in this study. Focusing on the specific location of primary tumors and bone metastatic sites, clinicopathological variables were included in a common database and analyzed retrospectively. Skeletal metastases were diagnosed according to the contemporary diagnostic guidelines and confirmed by bone scintigraphy. Besides region- and side-specific localization, primary tumors were also classified as central or peripheral tumors based on their bronchoscopic visibility.

**Results:** The most common sites for metastasis were the spine (*n* = 103) and the ribs (*n* = 60), followed by the pelvis (*n* = 36) and the femur (*n* = 22). Importantly, femoral (*p* = 0.022) and rib (*p* = 0.012) metastases were more frequently associated with peripheral tumors, whereas centrally located LADCs were associated with humeral metastases (*p* = 0.018). Moreover, we deduced that left-sided tumors give rise to skull metastases more often than right-sided primary tumors (*p* = 0.018). Of note, however, the localization of the primary tumor did not significantly influence the type of affected bones. Multivariate Cox regression analysis adjusted for clinical parameters demonstrated that central localization of the primary tumor was an independent negative prognostic factor for overall survival (OS). Additionally, as expected, both chemotherapy and bisphosphonate therapy conferred a significant benefit for OS.

**Conclusion:** The present study demonstrates unique bone-specific metastasis patterns concerning primary tumor location. Peripherally located LADCs are associated with rib and femoral metastases and improved survival outcomes. Our findings might contribute to the development of individualized follow‐up strategies in bone-metastatic LADC patients and warrant further clinical investigations on a larger sample size.

## Introduction

Lung adenocarcinoma (LADC) is the most frequent histologic subtype of lung cancer in both men and women (comprising between 40 and 50% of lung cancer cases worldwide) (1, 2). Since most LADCs are discovered at an advanced stage with different types of distant organ metastases already present, LADC remains one of the dominant causes of cancer-related deaths in Western countries (3, 4).

Extrathoracic metastases in LADC most commonly appear in the skeletal system. About 25–40% of all advanced-stage LADC patients acquire bone metastases throughout the course of disease progression (5–7) and are often complicated by skeletal-related events (SREs), causing pain and decreasing the patients’ functional and emotional well-being (5). Moreover, the presence of bone metastases is affiliated with shortened overall survival (OS) [< 1 year from diagnosis], significant morbidity, and increased social costs due to medical care (8). Notably, survival outcomes might also be influenced by the site of bone metastases (appendicular bone involvement is a negative prognosticator) (9). Therefore, it is apparent that early diagnosis and prevention of bone metastases is a relevant clinical issue in increasing the survival rate and quality of life of these patients. With regards to specific risk factors, the occurrence of skeletal metastases is significantly higher in patients with LADC histology compared to all other lung cancer subtypes (10). In addition, increased CA-125 and alkaline phosphatase (ALP) concentrations are also suggestive for skeletal metastases, and their serum levels correlate with the number of bone metastases sites (5). The existing treatment options for osseous metastases to date are pain relief, local radiation therapy (RTx), bone metabolism improvement, and radionuclide therapy (11). In this context, osteoclast inhibition using bone targeting agents (also called antiresorptive agents) such as bisphosphonates, is currently a rising topic in bone metastatic LADC (10, 12, 13). These synthetic pyrophosphate analogs inhibit osteoclast maturation and function, and induce osteoclast apoptosis (6, 14). They additionally slow down proliferation of osteoblasts and stimulate bone formation and differentiation (14). Hence, bisphosphonate therapy (BTx) is the standard care for the prevention and treatment of skeletal-related complications of bone metastases. Besides BTx, RTx is also an integral factor in the palliation of bone metastases (15, 16). RTx is performed primarily for pain relief, control of a bone affected by metastases and prevention of pathologic fractures (15, 16). Of note, oncogenic driver mutations should be taken into consideration during the administration of BTx and RTx since the beneficial effects of both therapeutic modalities might be less pronounced in patients with KRAS mutant tumors (17).

Even though distant organ metastases correlate highly with an unfavorable outcome in LADC patients, there has been no extended analysis concerning metastatic patterns and their effect on survival with regards to primary tumor location (i.e., bronchoscopic, side-specific and region-specific). Our group previously found that bone metastases were more common in patients with central tumors whereas patients with peripheral LADCs (7) presented more lung metastases. In addition, central LADCs were also associated with early metastatic spread (7). Yet, to date, the bone metastasis pattern is still largely unexplored in patients with exclusively bone metastases. Therefore, the intention of our cross-sectional study was to explore the influence of primary tumor location on bone metastasis site and type of affected bones as well as survival in a comprehensively large group of advanced-stage LADC patients with skeletal metastases. This information may provide insight for early surveillance guidance for bone metastasis detection or intervention in high-risk groups that improve the patients’ survival and quality of life.

## Patients and Methods

### Study Population

Consecutive patients with newly diagnosed advanced-stage LADC and synchronous bone metastasis were included in this study. All included patients were diagnosed, treated and followed-up at the National Koranyi Institute of Pulmonology, Budapest, Hungary and at the Department of Pulmonology of the Semmelweis University, Budapest, Hungary between September 1999 and August 2014. Cytological or histological examination was performed for patient diagnosis and bronchoscopic diagnostic procedures were applied on all patients. Bronchoscopically visible primary tumors were considered as centrally located LADCs, whereas endoscopically undetectable lesions were defined as peripheral tumors (7, 18). Regarding region-specific tumor localization on the right side, tumors in the upper and middle lobes were classified as upper region tumors whereas those in the lower lobe were termed lower region tumors (7). Similarly, LADCs in the left upper lobe (and lingula) were considered upper region tumors while those in the left lower lobe were classified as lower region LADCs (7). Bone metastases were identified by bone scintigraphy, computed tomography (CT) scan, PET-CT scan, magnetic resonance imaging (MRI) of the skeleton or standard X-ray. After the initial diagnosis of bone metastases, whole-body scintigraphy was performed in all cases to detect any additional skeletal metastases and confirm the predefined diagnosis. During the follow-up period, bone scans were performed on a regular basis every 3–4 months in accordance with the ESMO Clinical Practice Guidelines (19). The type of the affected bone was determined using guidelines of the National Institutes of Health (NIH) of the U.S. government (20). Accordingly, the following three major bone types were distinguished: long, flat, and irregular bones (of note, no bone metastases in short bones were identified in our study) (20). No other distant organ metastases were identified in the included patient cohort at the time of bone metastasis diagnosis. Clinicopathological data were collected retrospectively from the medical records and electronic patient record systems of the two participating institutions. Patients’ clinical data were handled anonymously and stored in a common, password-protected database with access only for investigators of this study. Importantly, in order to ensure comparability between the two centers, all medical records were reviewed case by case by an independent clinician. Based on the study aims, the parameters were assessed as follows: gender (dichotomous), smoking status (nominal), age at lung cancer diagnosis (continuous, interval), BTx (dichotomous), chemotherapy (CTx) [dichotomous], bronchoscopic-, side-specific and region-specific localization of the primary tumor (ordinal), and localization of bone metastases at diagnosis and during disease progression (ordinal). The 7^th^ edition of the TNM staging system was used to (re)classify all patients at the time of diagnosis. OS (provided by the Hungarian Central Statistical Office) was calculated from the initial diagnosis of bone metastasis (irrespective of the used imaging approach) until death of any cause or last available follow‐up visit. Clinical follow‐up terminated on the March 1, 2021.

### Treatment

All therapeutic approaches were conducted in accordance with the contemporary National Comprehensive *Cancer* Network (NCCN) guidelines (21) presenting no differences across the host institutes. CTx consisted of platinum-based combination CTx with either paclitaxel and carboplatin (PC), etoposide and cisplatin (EC) or gemcitabine and cisplatin (GC). Of note, targeted therapeutic agents became part of the standard of care therapy at the beginning of 2015 in Hungary. Therefore, only a small fraction of all included patients could have received targeted agents in the study interval. Regarding BTx, patients were treated either with first-generation bisphosphonate clodronate, second-generation bisphosphonate pamidronate or zoledronic acid given intravenously in 4-weeks cycles. In order to prevent pathological bone fractures and other SREs, palliative external beam RTx was also applied in some cases. None of the included patients received locoregional RTx in our study.

### Ethics Statement

The present study met the guidelines of the Helsinki Declaration (revised in 2013) of the World Medical Association. The national level ethics committee approved the study (Hungarian Scientific and Research Ethics Committee of the Medical Research Council, ETT‐TUKEB 23636–2/2018, 23 ,636/10/2018/EÜIG). The requirement for written informed consent was waived because of the retrospective nature of the study. All variables and data of interest were extracted from the institutional patient management systems and registered in a password-protected database. Patient identifiers were deleted after clinical information was extracted, disabling direct or indirect patient identification.

### Statistical Analyses

Patients were grouped according to the localization of their primary tumor and type/site of bone metastases. The Kolmogorov-Smirnov test was used to decipher the normality of data distribution. Categorical and ordinal parameters consisting of basic patient information, primary tumor localization and bone‐specific metastatic pattern were statistically analyzed by χ2 test or Fisher’s exact test. Risk assumptions were presented as odds ratio (OR) and respective 95% confidence intervals (CI). Kaplan–Meier plots were used to estimate survival curves and the log-rank test was applied to compare discrepancies between the groups. Median follow-up time was estimated using the reverse-censored Kaplan-Meier method with the R package prodlim. The independent prognostic value of the clinicopathological variables was examined with a multivariate Cox proportional hazard regression model. *p* values are always presented as two-sided and were considered statistically significant when below 0.05. Metric data is always shown as median or mean and corresponding range or, in case of OS, as median and corresponding 95% CI. All statistical analyses were performed using R version 3.6.3 (R Foundation for Statistical Computing, Vienna, Austria) and SPSS Statistics 23.0 package (SPSS Inc., Chicago, IL, United States).

## Results

### Patient Characteristics and Metastatic Sites

209 LADC patients with bone metastases were enrolled in this study. Their respective clinicopathological characteristics are summarized in Tables 1 and 2. The median age was 62  years (range 34–84). All patients had Caucasian ethnicity and 113 of them were male (54%) (Table 1). Peripheral tumors occurred more frequently than centrally-located tumors (59 vs. 41%). Right-sided LADCs were found in 57% (vs. left-sided, 43%) and upper region tumor location in 70% (vs. lower region 30%) of the patients. Primary tumor location did not produce significant variances in general clinicopathological characteristics. The most common metastatic sites for the localization of metastases (Table 2) were the spine (*n* = 103), the ribs (*n* = 60), the pelvis (*n* = 36), and the femur (*n* = 22), followed by humeral (*n* = 17), skull (*n* = 13), sternal (*n* = 10), and clavicular or scapular (*n* = 10) metastases. We identified 163 patients with metastases in one only one bone and 46 patients with multiple-bone metastatic disease at the time of diagnosis. Concerning specific bisphosphonate agents, 67, 29 and 57 patients received clodronate, pamidronate and zoledronic acid, respectively. Of note, there was no available data on the specific type of administered bisphosphonate agent in 15 patients’ cases). Palliative external beam RTx was applied in the case of 66 patients. Regarding major comorbidities, 53 individuals had COPD whereas hypertension was detected in 117 patients.

**TABLE 1:** Patient characteristics and tumor localization in LADC patients with consecutive bone metastases.

All patients		Localization of the primary tumor											
		Central	Peripheral	N/A	P value^a^	Left-sided	Right-sided	N/A	P value^a^	Upper or middle lobe^b^	Lower lobe	N/A	P value^a^
**Age (years)**													
<65	125	55	68	2	0.196^c^	52	69	4	0.986^c^	85	30	10	0.200^c^
≥65	84	30	54	0		36	48	0		53	28	3	
**Gender**													
Male	113	44	68	1	0.572^c^	51	60	2	0.342^c^	80	27	6	0.142^c^
Female	96	41	54	1		37	57	2		58	31	7	
**Smoking history**													
Never smoker	23	8	15	0	0.332^c^	10	11	2	0.652^c^	14	6	3	0.852^c^
Ex-smoker	50	25	25	0		19	31	0		37	12	1	
Current smoker	66	26	40	0		30	36	0		45	18	3	
N/A	70	26	42	2		29	39	2		42	22	6	

a *p* values refer to differences between patient characteristics and tumor localization.

b In the right lung: upper and middle lobes; in the left lung: upper lobe and ligula.

c χ^2^ test.

**TABLE 2:** General clinical characteristics of different metastatic sites in bone-metastatic LADC patients.

All patients	Bone metastasis site							
	Clavicle or scapula	Sternum	Skull	Humerus	Femur	Pelvis	Rib	Spine
**Total** ^a^	10	10	13	17	22	36	60	103
**Age (years)**								
<65	6 (60.0%)	7 (70.0%)	5 (38.5%)	13 (76.5%)	16 (72.7%)	21 (58.3%)	36 (60.0%)	58 (56.3%)
≥65	4 (40.0%)	3 (30.0%)	8 (61.5%)	4 (23.5%)	6 (27.3%)	15 (41.7%)	24 (40.0%)	45 (43.7%)
**Gender**								
Male	9 (90.0%)	6 (60.0%)	4 (30.8%)	12 (70.6%)	10 (45.5%)	17 (47.2%)	39 (65.0%)	52 (50.5%)
Female	1 (10.0%)	4 (40.0%)	9 (69.2%)	5 (29.4%)	12 (54.5%)	19 (52.8%)	21 (35.0%)	51 (49.5%)
**Smoking history**								
Never smoker	2 (20.0%)	2 (20.0%)	3 (23.1%)	1 (5.9%)	3 (13.6%)	4 (11.1%)	5 (8.3%)	15 (14.6%)
Ex-smoker	1 (10.0%)	3 (30.0%)	3 (23.1%)	3 (17.6%)	4 (18.2%)	11 (30.6%)	17 (28.3%)	21 (20.4%)
Current smoker	3 (30.0%)	0 (0.0%)	2 (15.4%)	7 (41.2%)	7 (31.8%)	10 (27.8%)	20 (33.3%)	30 (29.1%)
N/A	4 (40.0%)	5 (50.0%)	5 (38.5%)	6 (35.3%)	8 (36.4%)	11 (30.6%)	18 (30.0%)	37 (35.9%)

a The final number of included patients is 209. However, as a single patient does not necessarily present metastasis in specifically one bone, the overall number of metastases might be higher.

### Primary Tumor Location Is Linked to the Bone-Specific Site of Metastasis

**FIGURE 1:**
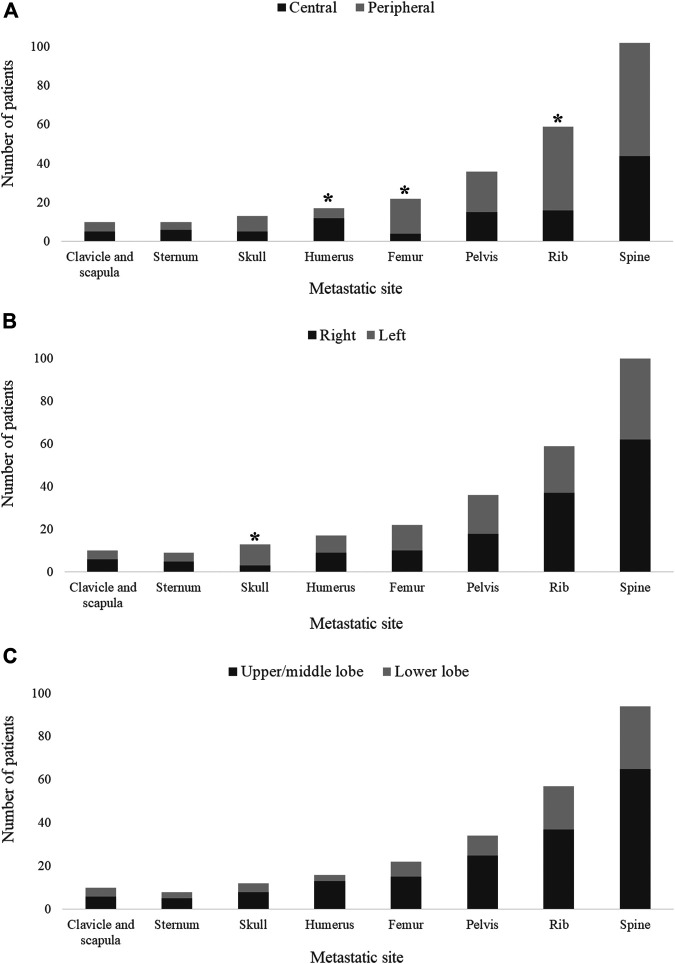
Primary tumor location and metastatic site in bone-metastatic LADC patients. **(A)** Peripherally located primary tumors are associated with femoral (OR 3.486, 95%CI 1.09–14.71, *p* = 0.022) and rib (OR 2.338, 95%CI 1.16–4.86, *p* = 0.012) metastases, whereas central LADCs give rise to humeral metastases (OR 0.262, 95%CI 0.06–0.83, *p* = 0.018). **(B)** Left-sided tumors are more frequently associated with skull metastases compared to right-sided primary LADCs (OR 4.836, 95%CI 1.19–28.19, *p* = 0.018). **(C)** No significant differences were found in metastasis pattern with regards to upper/middle lobe vs. lower lobe classification.

Exploring the ramifications of the primary tumor localization on the site of metastasis, we found that femoral (OR 3.486, 95%CI 1.09–14.71, *p* = 0.022) and rib (OR 2.338, 95%CI 1.16–4.86, *p* = 0.012) metastases were more frequently associated with peripheral tumors (Figure 1A) whereas centrally located LADCs were associated with humeral metastases (OR 0.262, 95%CI 0.06–0.83, *p* = 0.018; Figure 1A). Importantly, we additionally discovered that left-sided tumors more frequently give rise to skull metastases than right-sided primary tumors (OR 4.836, 95%CI 1.19–28.19, *p* = 0.018; Figure 1B). These results stayed significant at a 0.05 significance level using Bonferroni correction. Of note, there was no significant correlation between the primary LADC region (i.e., lower vs. upper region tumors) and the bone-specific metastatic site (Figure 1C). With regards to the type of affected bones, metastases in flat bones were more commonly found in patients with peripheral tumors (vs. central LADCs), yet these results were not statistically significant (*p* = 0.202; Supplementary Figure S1A). Likewise, as shown in Supplementary Figures S2A,B, the side- and region-specific primary tumor location did not influence the type of bone metastases either. The localization of the primary tumors did not have an impact on the number of metastatic bones (i.e., single-vs. multiple-bone metastatic spread) at diagnosis (data not shown).

### Prognostic Parameters and Clinical Outcome

**FIGURE 2:**
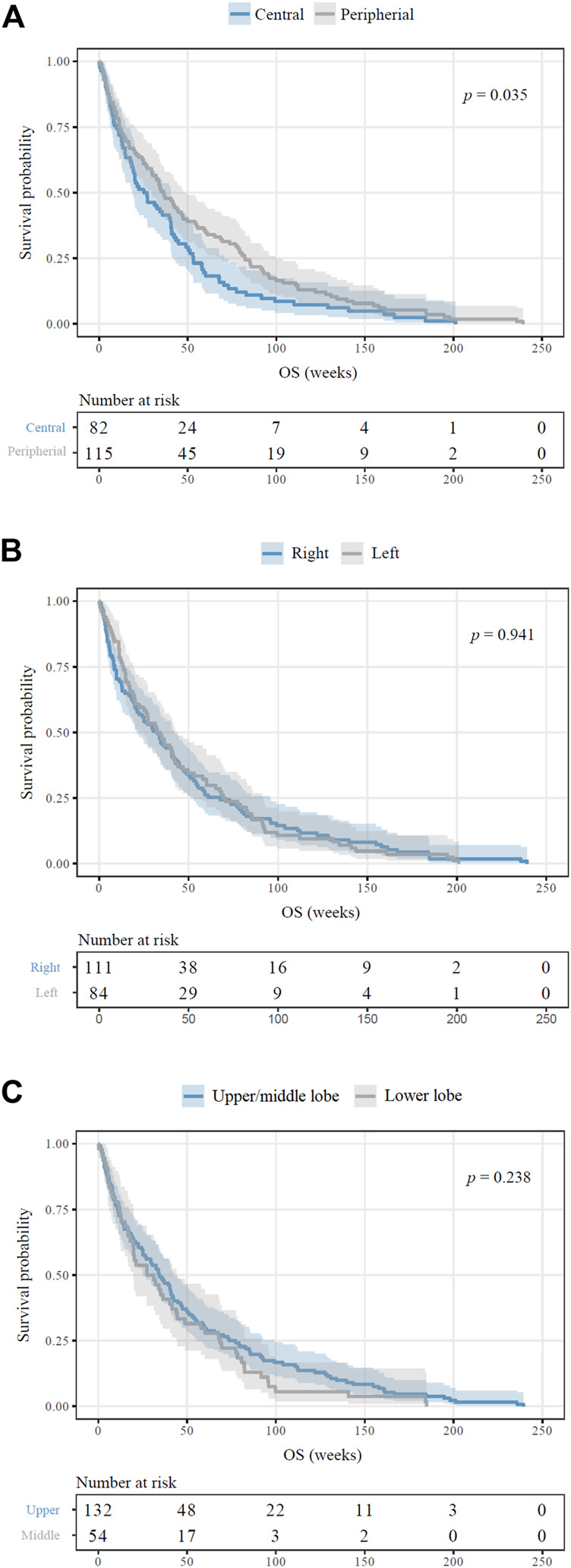
Survival outcomes of bone-metastatic LADC patients according to primary tumor location. **(A)** Patients with centrally located primary LADCs displayed significantly inferior OS in comparison to those with peripheral tumors (median OSs were 25.1 vs. 36.2 weeks, respectively; HR 1.359, *p* = 0.035). **(B)** Side-specific tumor localization did not have any impact on OS (*p* = 0.941). **(C)** There are no observed significant differences in OS for upper/middle vs. lower lobe (*p* = 0.238).

**FIGURE 3:**
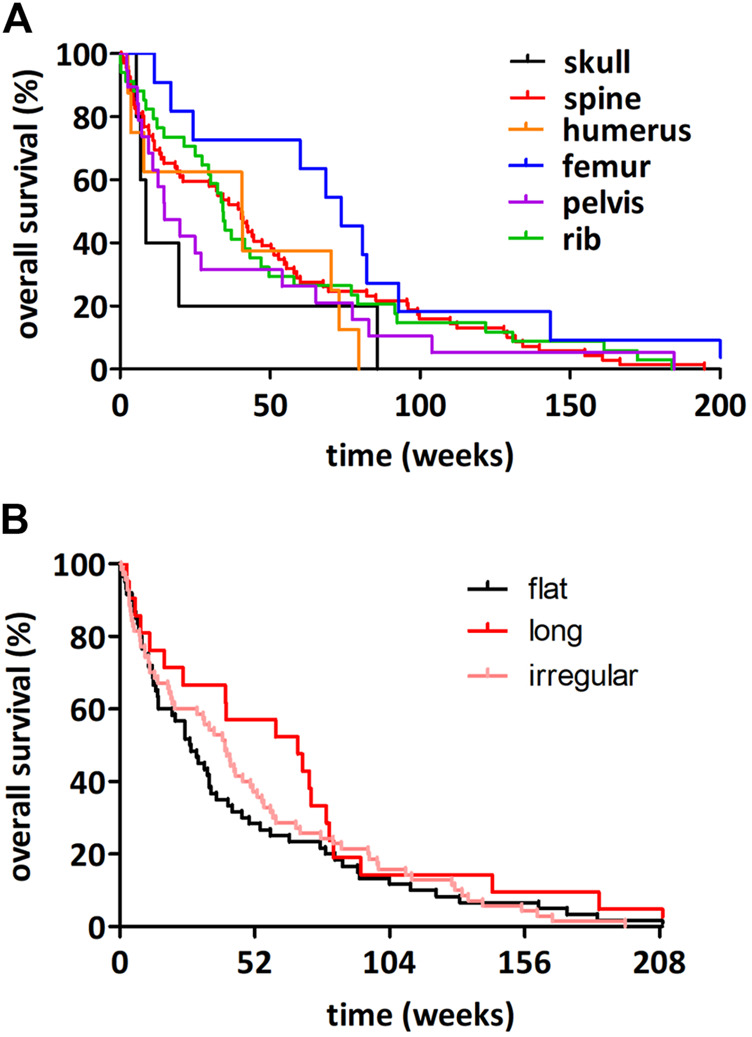
Kaplan-Meier plots for OS in LADC patients with solitary bone metastases according to affected bones. **(A)** The site of bone metastases did not influence the OS significantly (*p* = 0.307). **(B)** LADC patients with bone metastases in long bones have non-significantly longer median OS (vs. irregular bone vs. flat bone metastatic patients; median OSs were 68.5 vs. 40.4 vs. 27 weeks, respectively; *p* = 0.269).

The average follow‐up time for the entire cohort consisting of 209 bone-metastatic LADC patients was 33.7 weeks (of note, survival data was not available in the case of 10 patients). Outcome of survival was worse for patients whose primary LADCs were located centrally in comparison to patients presenting peripheral tumors (median OS, 25.1 vs. 36.2 weeks, HR 1.359, 95%CI 1.020–1.810, *p* = 0.035, Figure 2A). There were no significant distinctions in patients with right vs. left (*p* = 0.941; Figure 2B) or upper vs. lower region (*p* = 0.238; Figure 2C) located primary tumors concerning OS. We consecutively deduced whether there was an association between the number of metastatic sites and survival outcomes, but found that the number of affected bones did not influence the median OS (*p* = 0.436; data not shown). When comparing the survival outcomes of LADC patients with solitary bone metastases, we discovered that the site of bone metastases did not significantly influence survival (*p* = 0.307; Figure 3A). Importantly, however, patients with femoral metastases were more likely to have better survival outcomes than those with other bone metastases (*p* = 0.064; Supplementary Figure S2). Although the median OS was visibly longer in patients with bone metastases affecting long bones (vs. flat bones vs. irregular bones), this trend does not seem to be statistically significant either (*p* = 0.269; Figure 3B). With regards to specific therapeutic approaches, as expected, BTx-naive patients had significantly worse median OS than those receiving BTx (median OS, 12.0 vs. 40.2 weeks, HR 2.101, 95%CI 1.462–3.020, *p* < 0.001, Supplementary Figure S3A). Similarly, CTx also conferred a significant benefit for OS when compared to CTx-naive patients (median OS, 50.2 vs. 17.4 weeks, HR 0.545, 95%CI 0.410–0.726, *p* < 0.001, Supplementary Figure S3B). In order to assess if the prognostic value of tumor location (i.e., central vs. peripheral) was independent of other prognostic factors, we performed a multivariate Cox regression analysis (Table 3). Importantly, we found that there was a significant association between the peripheral location of primary LADCs and a benefit in OS (HR 0.589, *p* = 0.001, Table 3). Cox regression analysis confirmed our expectation that the specific therapeutic approaches (BTx and CTx) also independently influence survival outcomes (*p* < 0.001).

**TABLE 3:** Multivariate Cox Regression model for clinicopathological variables influencing the OS.

Clinicopathological variable	OS
**Localization (central vs. peripheral)**	
HR	0.589
95% CI	(0.438–0.794)
*p*	0.001
**BTx (no vs. yes)**	
HR	0.425
95% CI	(0.292–0.619)
*p*	<0.001
**CTx (no vs. yes)**	
HR	0.515
95% CI	(0.383–0.692)
*p*	<0.001

OS, overall survival; HR, hazard ratio; CI, confidence interval; BTx, bisphosphonate therapy; CTx, chemotherapy.

## Discussion

Patients with advanced-stage LADC presenting distant metastases most frequently develop them in the skeletal system (22). These skeletal metastases give rise to metabolic disorders such as pathologic fractures and spinal cord compression or hypercalcemia and therefore severely affect the quality of life and survival (5). Accordingly, bone metastases are generally affiliated with a poor prognosis with median survival rates of a couple months (6, 8, 15). Immediate and effective therapeutic strategies can moderate the severity of SREs and prolong patient survival (6). Therefore, identification of specific metastatic patterns and diagnostic markers is integral to enable clinicians to choose the appropriate treatment for each patient. The objective of this study was to assess the bone-specific metastasis pattern of advanced-stage LADC patients, and moreover to investigate how the precise primary tumor location affects survival and bone specificity.

In the current cohort of 209 LADC patients diagnosed with simultaneous bone metastases, we found that the most common site of skeletal metastases was the spine, followed by the ribs and pelvis. These results are consistent with previous findings of Tsuya et al. (23) who also observed that the spine was the most common site for metastasis (followed by the ribs). Of note, these vertebral metastases are of clinical importance since spinal metastases are commonly associated with pathological fracture, pain and collapse (24). While the exact mechanism for tumor invasion of the spine is still being researched, the presence of certain factors such as RANK and RANKL which interact with receptors to activate osteoclastic cells seems to play a pivotal role in setting an island of invasion (25, 26). Furthermore, another possible reason for the high incidence of spine and rib metastases in lung cancer patients might be the existence of venous traffic branches among lung, intercostal and vertebral veins, as well as the close proximity between these organs (5, 27).

The impact of primary tumor location on the site preference of organ metastases has been investigated in different solid tumors including non-small cell lung cancer (NSCLC) (7, 28, 29), small cell lung cancer (SCLC) (30), colorectal‐(31–33) and pancreatic cancer (34). In pancreatic cancer, primary tumors in the pancreatic body or tail correlated with a less aggressive phenotype compared to tumors in the head of the pancreas (34). As for colorectal cancer, tumors on the left side are associated with a greater metastasis rate than right-sided primary tumors (35, 36). With regards to lung cancer, our group previously found that peripheral LADCs more often give rise to lung metastases whereas skeletal metastases are more common in central tumors (7). In contrast, the metastasis pattern was not influenced by the bronchoscopic localization of the primary tumor in SCLC patients (30). In the present study, we found that femoral and rib metastases were more frequently associated with peripheral tumors whereas centrally located LADCs were associated with humeral metastases. Additionally, we demonstrate that left-sided tumors give rise to skull metastases more frequently than right-sided primary tumors. Of note, the incidence of both skull and humerus metastasis was relatively low in our cohort. Therefore, these results have to be cautiously interpreted until further validation. A possible explanation for the relationship between peripheral tumors and rib metastases might be the already mentioned venous traffic branches among lung periphery and intercostal veins, as well as the relatively short distance between lung periphery and the ribs (5, 27). Thus, the LADC cells in peripheral tumors can easily spread to the ribs through both hematogenous dissemination and direct invasion. Interestingly, we also found that left-sided tumors were more frequently associated with skull metastases than right-sided LADCs. In contrast, however, the region-specific tumor localization (i.e., upper and middle lobe vs. lower lobe tumors) did not influence the metastasis pattern. Since treatment options for patients bearing bone metastases might vary depending on the type of the affected bones, we also assessed the influence of primary tumor location on the bone-type specific metastasis pattern (37). In our cohort, metastases in flat bones were more common in patients with peripheral LADCs compared to central tumors, yet these results were not statistically significant. Importantly, however, this tendency might still be of therapeutic relevance since therapeutic procedures via radiofrequency ablation are hard to implement for metastases affecting flat bones (37). To date, the exact pathophysiological mechanisms that lie behind the bone-specific metastasis patterns are largely unexplored. Besides the already mentioned vasculature-specific features, another explanation might be related to the differences concerning the histological subtype and mutational status of the primary LADCs (38, 39). Nevertheless, neither the histological subtype nor the mutation status of the primary tumor were available in our study.

Variances in cancer mortality between primary tumors located on different sides have been noted in several studies. Right-sided localization turned out to be an independent negative predictor of survival in patients with metastatic colorectal cancer, yet the localization of the primary tumor did not influence the OS in stage II and III patients (40, 41). In breast cancer, the primary tumor location also influences survival outcomes as upper-outer quadrant breast tumors present a more favorable survival advantage (compared to tumors in other locations) (42). Our results revealed that decreased median OS of patients followed after central primary LADC localization compared to peripheral tumor location. Furthermore, we also obtained that the endoscopic localization of the primary tumor predicted outcome independently of other variables. This finding is in accordance with existing data also suggesting that central primary tumor location is associated with significantly worse survival outcomes both in early- and advanced-stage lung cancer (7, 43, 44). Yet, this study is the first to investigate the effect of primary tumor location in LADC patients with bone-only metastases. Nevertheless, our data suggest that differences in survival vary neither between left- and right-sided LADCs, nor between upper- and lower region tumors. Hence, these tumors might not comprise separate entities and should be handled with the same oncological principles. These outcomes are supported by the results of a large Surveillance Epidemiology and End Results (SEER) analysis where the prognosis between right- and left-sided NSCLC in stage I–IIIA was similar, irrespective of whether patients had surgery performed or not (45). Furthermore, *Puri et al.* (46) and Whitson *et al.* (47) also concluded that the side- and region-specific primary tumor localization does not influence survival in early-stage patients. With regards to the prognostic significance of bone metastasis localization and type of affected bone, we found that metastases in long bones (and particularly in the femur) are associated with longer median OS compared to other bone metastases. Meanwhile, metastases in flat bones (i.e., skull) were associated with impaired survival outcomes. Even though these results were not statistically significant, a tendency warranting further investigation can be observed. A possible explanation of impaired survival in patients with metastases in flat bones might be that these metastases can occupy the adjacent anatomic structures more easily. Accordingly, calvarial metastases might invade into dura and intradural space, causing meningeal irritation, increased intracranial pressure, and focal neurological signs ultimately leading to worse survival (48). Moreover, as mentioned previously, RTx is more difficult to enact for metastases affecting flat bones whereas femoral bone metastases are relatively easy to target via radiofrequency ablation (37).

Although we successfully explored the bone-specific metastasis pattern and the associations between primary tumor localization and metastasis site in LADC patients, some study limitations remain. First, the retrospective nature and the lack of a validation set limit this study. It is therefore apparent that results of the present study have to be interpreted with caution and some details need to be confirmed in a prospective setting. Second, although our cohort was homogenous in terms of histology, disease stage, ethnicity and site of metastasis (exclusively bone metastases), our strict inclusion criteria greatly reduced the sample size of included patients. Therefore, subgroup analyses with regards to the type of spinal metastases could not be performed. Additionally, given the relatively low number of cases with certain metastases (i.e., skull and humerus), our results concerning the metastasis pattern of these bones require further independent studies. Of note, although diagnostic procedures such as whole-body scintigraphy were performed on a regular basis during the follow-up period, the risk of underdiagnosis of certain bone metastases should be also taken into account. Third, no information on the exact dose and cycles of the administered CTx, BTx and RTx was available. This might also affect survival outcomes. An additional limitation was the absence of detailed clinicopathological data regarding disease history, other co-morbidities and tumor characteristics (including its KRAS mutational status). Of note, KRAS mutation was found to be a negative prognosticator in these patients. Therefore, KRAS mutational status should be considered when making decisions in bone metastatic LADC patient therapy (17). In addition, according to the “seed and soil” hypothesis, the histological subtype and the mutational status of the primary tumor might also have an impact on the specific metastasis patterns (38, 39). Lastly, the methodology used to separate the primary tumors into central and peripheral lesions via bronchoscopic visibility might lead to unbiased decision making for some results. To date, however, there are no standard definitions for centrally vs. peripherally located lung tumors (7). Considering these study limitations, it is necessary to take caution during the interpretation of our results and further prospective studies are warranted.

In conclusion, as far as we know, this is the first study investigating the impact of primary tumor location on bone-specific metastasis pattern in advanced-stage LADC patients. Our results indicate that femoral and rib metastases are more frequently associated with peripheral tumors whereas centrally located LADCs are associated with humeral metastases. Furthermore, we show that patients with central LADCs present worse OS than patients with peripheral tumors. Altogether, our findings might facilitate early diagnosis and contribute to the development of individualized treatment plans and follow‐up strategies in bone-metastatic LADC patients. Importantly, due to the relatively low number of included patients and the abovementioned study limitations, our study is rather hypothesis-generating than confirmatory. Therefore, further studies on independent cohorts are warranted to confirm the bone-specific metastasis pattern in these patients.

## Data Availability

The raw data supporting the conclusions of this article will be made available by the authors, without undue reservation.

## Supplementary Material

Supplementary Figure 1 Primary tumor localization and type of affected bone. **(A)** The percentage of LADC patients with metastases in flat bones (vs. other bone type) was non-significantly higher in case of peripheral tumors (vs. central LADCs; *p* = 0.202). **(B,C)** The side- and region-specific localization of the primary tumor did not influence the type of bone metastases.

Supplementary Figure 2 Survival outcomes of LADC patients with femoral metastases. Patients with femoral metastases exhibited non-significantly superior OS in comparison with those with other bone metastases (median OSs were 73.7 vs. 33.7 weeks, respectively; *p* = 0.064).

Supplementary Figure 3 Kaplan-Meier estimates for OS according to specific therapeutic approaches. **(A)** BTx-naive patients had decreased median OS (vs. BTx-treated individuals; median OSs were 12.0 vs. 40.2 weeks, respectively; HR 2.101, *p* < 0.001). **(B)** Patients treated with CTx had significantly longer OS than those not receiving CTx (median OSs were 50.2 vs. 17.4 weeks, respectively; HR 0.545, *p* < 0.001).
