# Supplementary figures and images for: Bone-Specific Metastasis Pattern of Advanced-Stage Lung Adenocarcinoma According to the Localization of the Primary Tumor
Source: Pathol Oncol Res. 2021 Sep 23;27:1609926. doi: 10.3389/pore.2021.1609926 (PMC8496061; doi:10.3389/pore.2021.1609926)

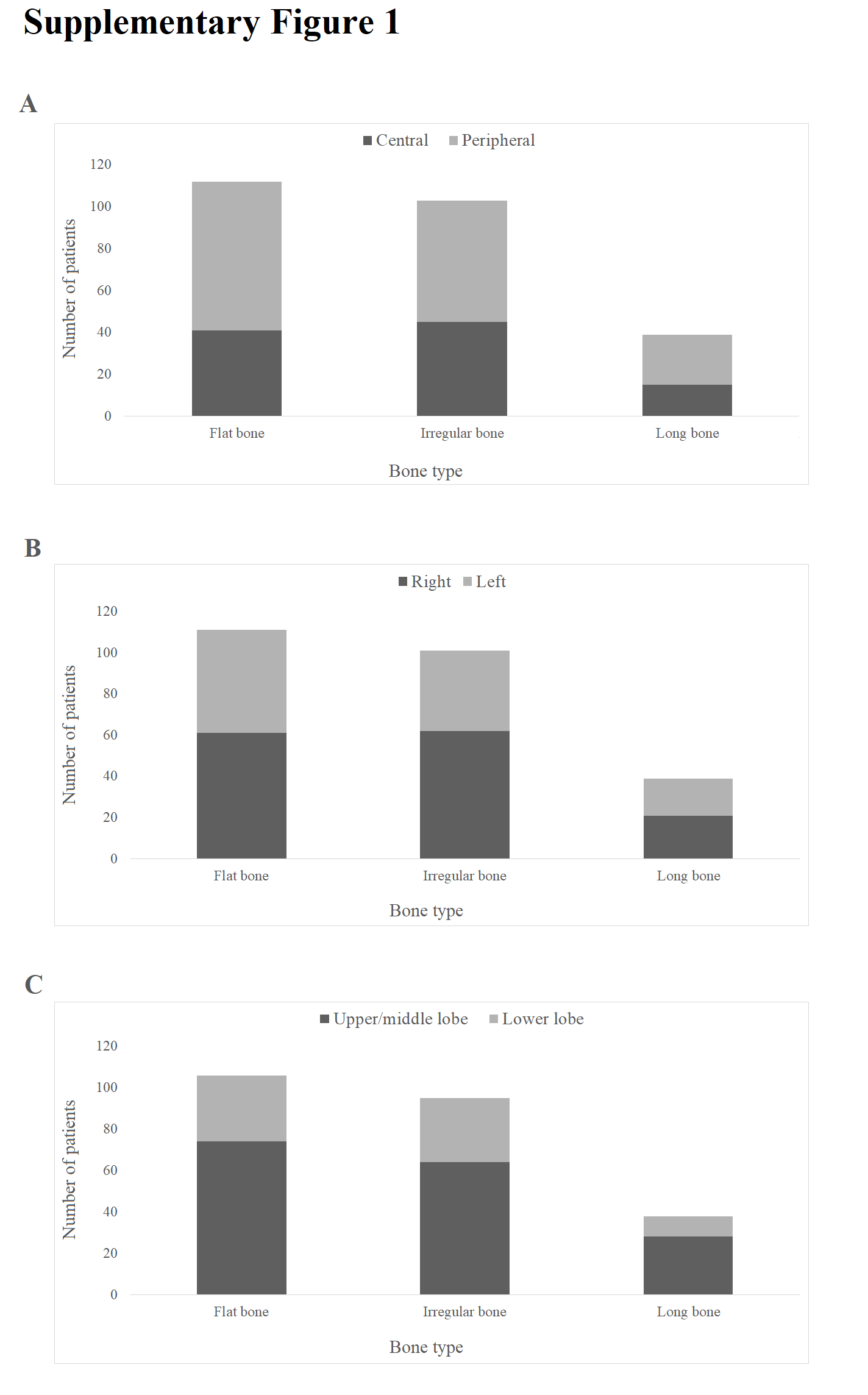

Supplement: Supplementary file 1 [file Image1.TIF]

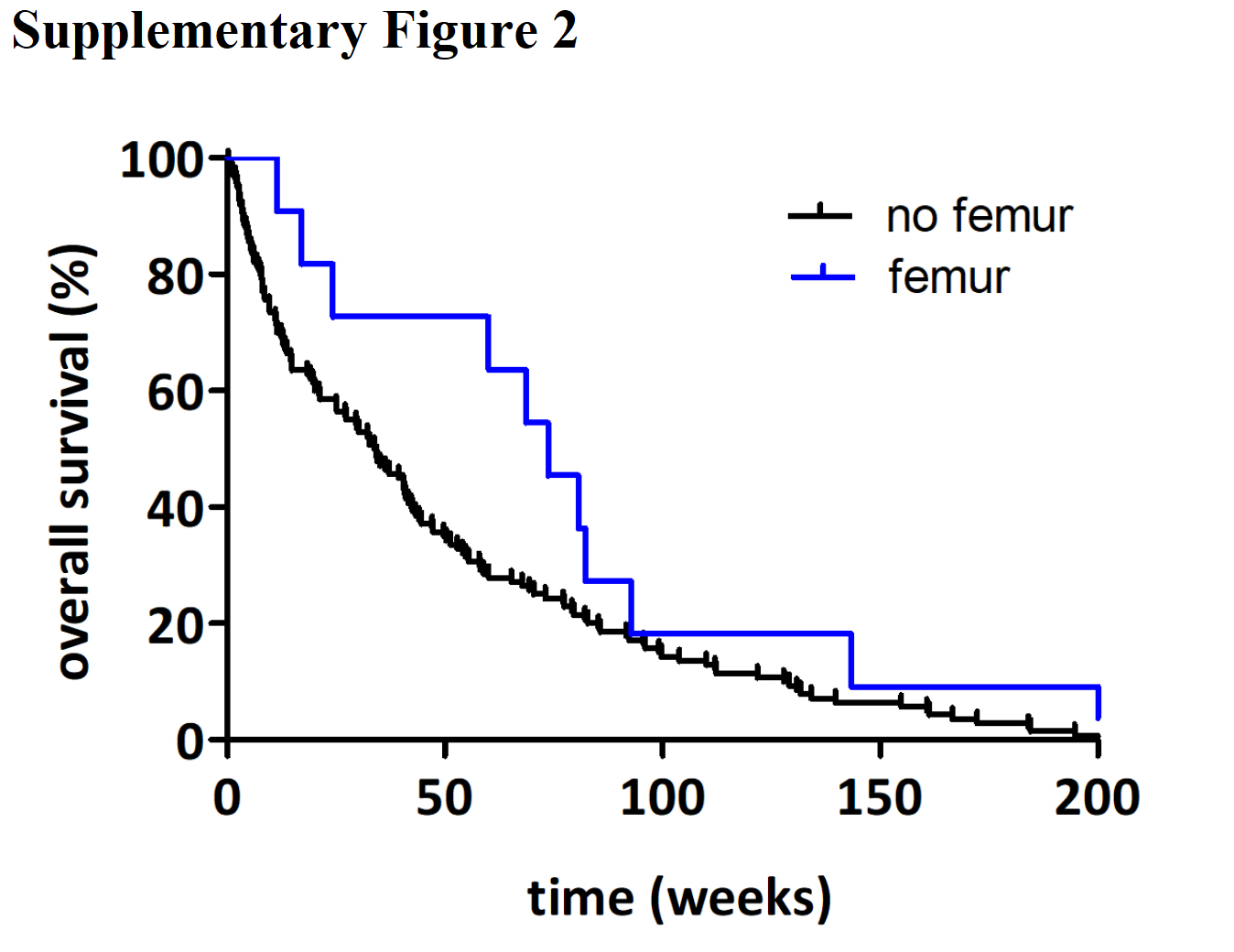

Supplement: Supplementary file 2 [file Image2.TIF]

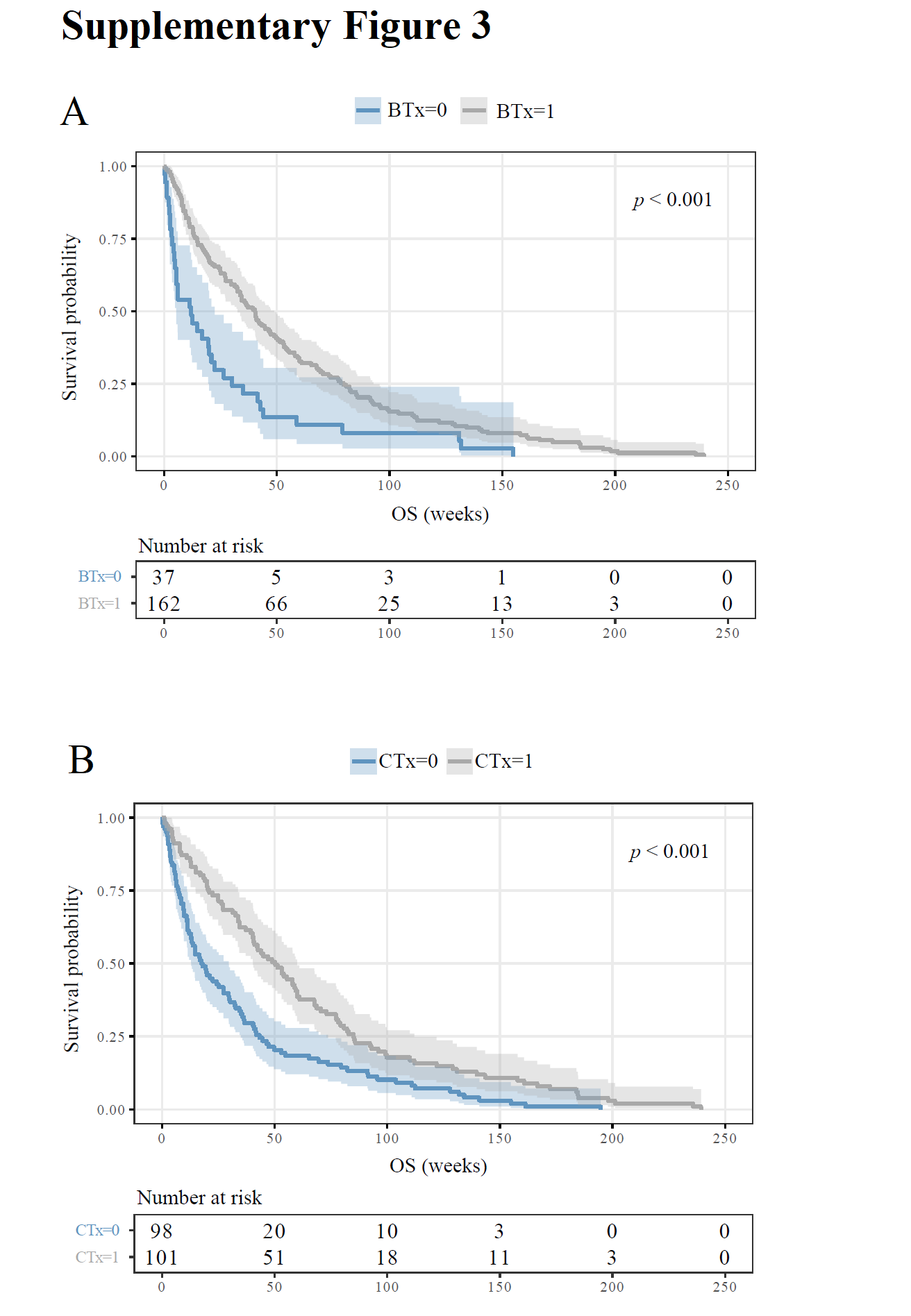

Supplement: Supplementary file 3 [file Image3.TIF]
